# Anomaly detection as a tool for identifying cases of interest in postmortem toxicology

**DOI:** 10.1038/s41598-026-65664-5

**Published:** 2026-08-17

**Authors:** Carl Söderberg, Oleg Sysoev, Rasmus Magnusson, Fredrik C. Kugelberg, Henrik Green

**Affiliations:** 1 Department of Forensic Genetics and Forensic Toxicology, National Board of Forensic Medicine, Artillerigatan 12, 587 58 Linköping, Sweden; 2 Division of Statistics and Machine Learning, Department of Computer and Information Science, Linköping University, 581 83 Linköping, Sweden; 3 Department of Biomedical Engineering, Linköping University, 581 83 Linköping, Sweden; 4 Division of Clinical Chemistry and Pharmacology, Department of Biomedical and Clinical Sciences, Linköping University, 581 83 Linköping, Sweden; 5 Division of Clinical Chemistry and Pharmacology, Department of Biomedical and Clinical Sciences, Science for Life Laboratory, Linköping University, 581 83 Linköping, Sweden

**Keywords:** Postmortem toxicology, Fatal intoxication, Anomaly detection, Reference concentrations, Femoral blood, Chemistry, Drug discovery, Medical research

## Abstract

Intoxication-induced death, both intentional and unintentional, is a global public health concern, contributing substantially to mortality in many regions. Postmortem toxicological interpretation typically relies on reference concentrations of individual substances, which fails to account for combinatorial effects when multiple drugs are present. More comprehensive approaches that analyse multiple substances simultaneously could improve diagnostic accuracy. This study applied computational anomaly detection methods to identify toxicological profiles deviating from known non-intoxication patterns and assessed whether such deviations correspond to fatal intoxications or could serve as a screening tool for cases requiring further review. Postmortem femoral blood drug concentrations covering 191 substances from 16,710 forensic autopsies were analyzed. Cases were divided into a training set of non-intoxications (*n* = 8320) and separate validation (*n* = 2674) and test sets (*n* = 3342) containing both intoxications and non-intoxications. A machine learning–based anomaly detection model was trained and optimized before being applied to the test set. The model distinguished intoxications from normal patterns with 83.9% accuracy. False negatives often involved opioids and ethanol, while false positives were associated with rare substances, numerous detections, or high concentrations. Overall, this approach demonstrates strong potential as a screening tool for identifying toxicological patterns of forensic interest.

**Supplementary Information:**

The online version contains supplementary material available at 10.1038/s41598-026-65664-5.

## Introduction

Intoxication-induced death, both intentional and unintentional, is a serious and widespread issue. Drug-related deaths constitute a substantial proportion of these fatalities. In the US alone, 106,699 drug overdose deaths occurred in 2021^1^. Recent statistics from the EU indicate that illicit drug overdose comprise 2.25 deaths per 100,000 inhabitants in 2022^2^. Globally, unintentional poisonings comprised 0.7 deaths per 100,000 population in 2021^3^.

Correctly identifying cases of fatal intoxication involves the combined evaluation of numerous factors such as circumstantial information, autopsy findings and postmortem toxicological results. A key aspect, in order to identify potential cases of intoxication, is discerning if a pattern of substances and their concentrations can be considered normal or anomalous. However, correctly evaluating the impact of multiple substances is multifaceted and difficult^4–7^. Additionally, the evaluation of postmortem toxicological results is further complicated by unique postmortem factors, such as the postmortem redistribution of drugs, postmortem metabolism, and stability, by which concentrations change after death^8–10^.

To address these challenges, forensic practitioners have developed postmortem reference concentrations using two principal approaches. A Finnish series of studies has compiled substance detections across all causes of death, generating descriptive statistics for a large number of substances and providing robust reference material^11,12^. However, because all causes of death are pooled, this approach offers limited guidance on defining toxic concentrations. In contrast, a Swedish approach stratifies cases by cause of death, distinguishing controls from intoxications and providing concentration ranges for each group^13–18^. While this allows clearer interpretation of levels that may be contributory to death, it includes fewer substances, is based on smaller sample sizes, and may be affected by circular reasoning, as forensic diagnoses partly rely on the same concentration data.

Irrespective of approach, current postmortem reference compilations struggle to account for the effect of multiple pharmacologically relevant substances, i.e. the reference concentration does not adapt or change according to the presence of other substances. This is a serious constraint, since international studies indicate that fatal overdoses commonly involve multiple substances^19,20^. Consequently, complex and irregular interaction patterns involving multiple substances, each within established reference ranges, may not be identified by current reference concentrations and compilations. This limitation may lead to an underestimation of synergistic or cumulative toxic effects, thereby failing to uncover cases of importance.

However, there is currently no clear way to define an anomalous toxicological pattern with respect to multiple substances without introducing possible bias from current compilations of reference concentrations. One data driven way to approach this issue is to instead characterize the toxicological patterns found in non-intoxication deaths with the assumption that cases that have different toxicological patterns than seen in non-intoxication deaths are either probable intoxications or other cases with toxicological patterns of forensic importance. The scientific field of outlier/anomaly-detection focuses on methods for finding irregular patterns in data and have been used with success in fields such as medical diagnostics and bank security^21,22^. An advantage of these methods is that they can handle high-dimensional data^23^, which makes it possible to evaluate combinations of multiple substances at the same time. However, constructing a robust anomaly detection model requires access to extensive postmortem toxicological data.

In Sweden, the National Board of Forensic Medicine conducts between 5500 and 6000 autopsies annually. All toxicological analysis in these cases is performed by a central laboratory, the Department of Forensic Genetics and Forensic Toxicology, and the results are stored in a central database. The database contains all postmortem toxicological results from 1992 onwards comprising all causes of death, presenting a unique opportunity to leverage this large dataset for model building.

However, there is a wide selection of different models for anomaly detection in which performance can vary according to the type of expected anomaly and the type of the dataset^24–26^. To the best of our knowledge, anomaly detection models have not been applied to postmortem forensic toxicology. Consequently, there is no existing knowledge concerning which models and which model hyperparameters best fit this domain.

In the present proof-of-concept study, we utilize our large repository of postmortem toxicological concentration data to train an anomaly detection model using non-intoxication cases and to evaluate its potential as a screening tool in routine postmortem toxicological investigations across all causes of death, complementing existing reference compilations. Specifically, we assess the extent to which anomalous toxicological patterns correspond to cases currently classified as fatal intoxications, and whether the identification of such anomalies may highlight cases of forensic interest even in the absence of an intoxication diagnosis, thereby enabling the detection of potentially overlooked cases.

## Materials and methods

### Definition of terms

Herein, the term *intoxication* refers to a postmortem case in which the attending forensic pathologist has attributed an intoxication with drugs and/or alcohol as the primary cause of death (either immediate or contributing) or as a supporting cause of death. *Non-intoxication* refers to a case which lack an intoxication diagnosis as either a primary or supporting cause of death.

With regard to the correspondence between anomalous patterns and intoxications we define a *true positive* (TP) as a case with an anomalous toxicological pattern (according to the model) and which the forensic pathologist has diagnosed as an intoxication. Correspondingly, a *false positive* result (FP) is one where the toxicological pattern is anomalous while the attending forensic pathologist has diagnosed as a non-intoxication. A *true negative* result (TN) is a case with a normal toxicological pattern and which the forensic pathologist has attributed as a non-intoxication. A *false negative* result (FN) is a case with a normal toxicological pattern, but which the forensic pathologist has diagnosed as an intoxication.

The trained model’s *sensitivity* and *specificity* were defined normally as follows:

$${\text{Sensitivity = }}\frac{{\text{number of true positives}}}{{\text{number of true positives + number of false negatives}}}$$

$${\text{Specificity = }}\frac{{\text{number of true negatives}}}{{\text{number of true negatives + number of false positivies}}}$$

and the *positive predictive value* (PPV) and *negative predicted value* (NPV) were accordingly calculated as:

$${\text{PPV = }}\frac{{\text{number of true positives}}}{{\text{number of true positives + number of false positives}}}$$

$${\text{NPV = }}\frac{{\text{number of true negatives}}}{{\text{number of true negatives + number of false negatives}}}$$

### Study population

**Fig. 1:**
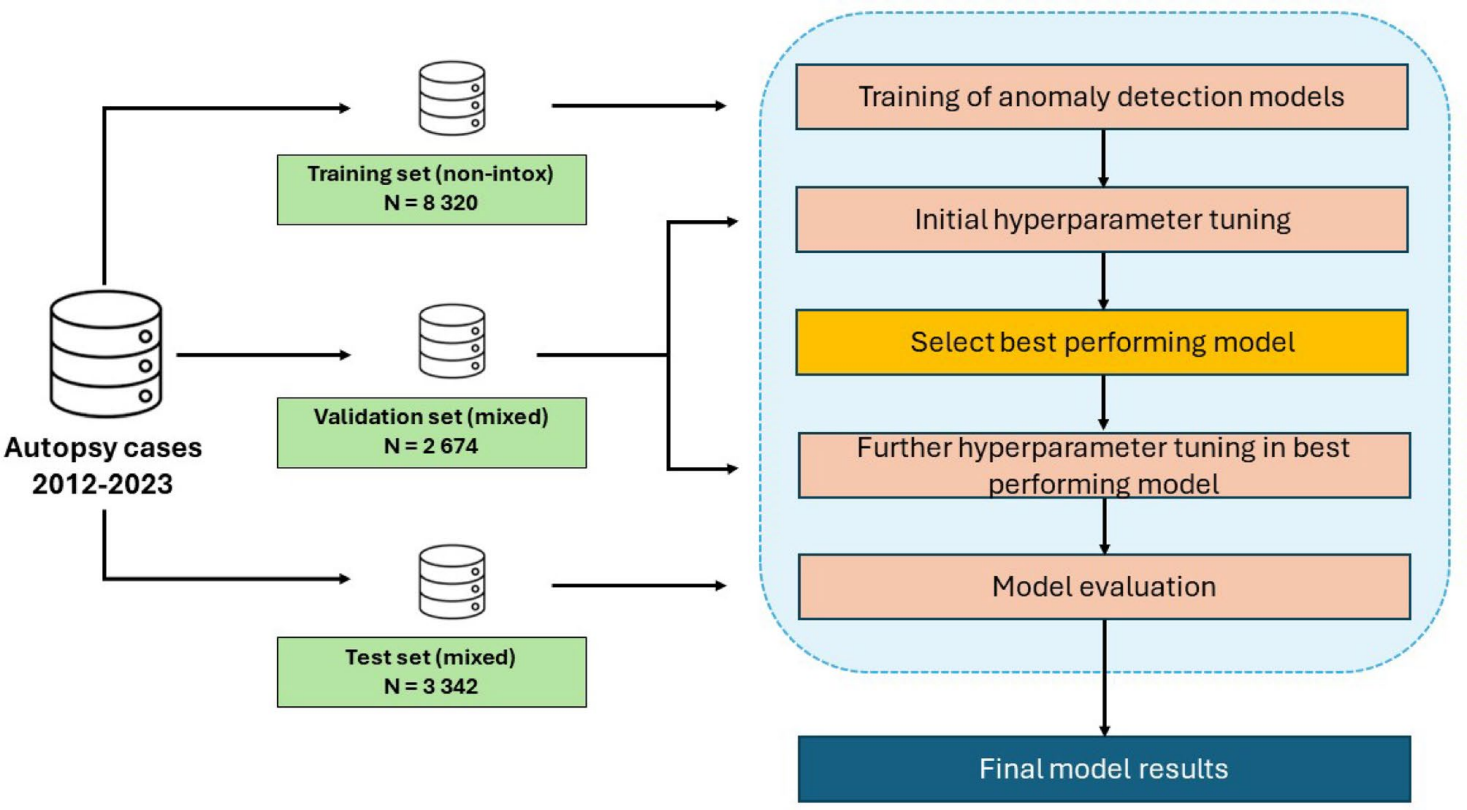
Overview of study design.

All forensic autopsy cases between 2012 and 2023 in which a toxicological analysis had been performed in femoral blood were included in the study (*n* = 58,933). Since an anomaly detection models evaluates patterns in the data, care was taken not to introduce a bias based on potential differences in analytical strategy between non-intoxication and intoxication cases. To this end, cases in which an analysis had been performed (with either a positive or negative result) outside the general screening methods and verification methods employed in all postmortem cases were excluded (*n* = 31,925). Additionally, out of the remaining eligible cases (*n* = 27,008), cases with an unknown cause of death, cases in which the potential impact of drugs or alcohol was unclear or included certain pathologies and intoxications (see supplemental Table 1), as well as cases with all negative findings were removed. In total 16,710 autopsy cases were included in the study, comprising 3722 (22%) intoxications and 12,988 non-intoxications (78%). An overview of the study design is presented in Fig. 1.

### Analytical methods

All postmortem cases were initially processed using a previously described standardized screening procedure in femoral blood using liquid chromatography/time-of-flight mass spectrometry (LC-TOF–MS)^27^. Positive detections were validated using targeted LC–MS-MS methods. Alcohols (ethanol, isopropanol and methanol) in femoral blood were analyzed by a headspace gas chromatography flame ionization detector (HS-GC-FID) method^28^.

### Preprocessing

Out of the whole dataset of 16,710 autopsy cases, 20% (*n* = 3342, of which 22% were intoxications) were randomly selected and set aside for use as a test set. Of the remaining cases (*n* = 13,368) 20% were set aside as a validation set for hyperparameter tuning (*n* = 2674, of which 23% were intoxications). The remaining cases (*n* = 10,694) were designated as potential training cases, for the final training set all intoxication cases (*n* = 2374) were removed leaving a training set comprising a total of 8320 cases (non-intoxications only) for the training of the anomaly detection model.

The complete dataset (*n* = 16,710) included 191 different substances as potential model dimensions. However, because of postmortem instability in the case of flunitrazepam and 7-amino-flunitrazepam, nitrazepam and 7-amino-nitrazepam, clonazepam and 7-amino-clonazepam, propiomazine and dihydropropiomazine^13,29,30^, concentrations of these substances were combined respectively. This adjustment left 187 dimensions for training the anomaly detection model.

Concentrations of alcohols (ethanol, isopropanol, methanol) were recorded in per mille (‰, g/kg). Buprenorphine, norbuprenorphine and fentanyl concentrations were recorded in ng/g. Concentrations of all other included substances were recorded in µg/g.

For each case concentrations of substances not found, i.e. below the analytical methods limit of reporting (LOR) and thus a negative result, was assigned a concentration of 0.0 µg/g (or ‰ or ng/g respectively).

### Initial model selection

For model selection and evaluation, we used the Python programming language (v.3.9.19) together with the package PyOD (v.2.01)^31^ for anomaly detection and the package Scikit-learn (v.1.4.2) for general tools.

In evaluating models, we used the area under the receiver operating characteristic curve (AUROC)^32^ as the primary performance metric. AUROC measures a model’s ability to distinguish between positive and negative classes across all possible classification thresholds, where a value of 0.5 indicates no discriminative ability (equivalent to random guessing) and a value of 1.0 indicates perfect discrimination. Thus, higher AUROC values reflect better overall classification performance.

For model selection we initially tested a subset of models each using different methodologies; Isolation Forest^33^, Deep Isolation Forest^34^, Deep One-Class Classification^35^ and an autoencoder^36^. Models were tested using a grid-search across a small set of hyperparameters and evaluated using the AUROC on the validation set. For the neural network models (autoencoder and the deep one-class classifier) the number of training epochs were equalized (*n* = 50). Default settings were used for the other hyperparameters. Performance was similar across models and hyperparameters, with the autoencoder model showing slightly better performance and stability than the other models (see supplemental Table 2). The autoencoder model was selected for further hyperparameter optimization.

### Autoencoder: reconstruction error and outlier score

In short, an autoencoder-based anomaly detection model uses a neural network to first reduce a dataset to a lower dimensional space and then reconstruct the original dataset from its lower dimensional representation. The training set is used to train the model to minimize the reconstruction error across all dimensions in the training set for each included case;

$${\text{reconstruction error}} = \mathop \sum \limits_{i = 1}^{d} \left( {x_{i} - x_{i}^{\prime} } \right)^{2}$$

where $x_{i}$ is the input concentration of substance *i*, $x_{i}^{\prime}$ is the reconstructed output and *d* is the number of dimensions^36^. This is done for all substances (dimensions), which in our case is 187 (the number of included substances, see above), and summarized for each case, resulting in the case specific reconstruction error.

Anomalous data points tend to have higher reconstruction errors due them having uncommon patterns compared to the training set used to train the model. The reconstruction error is then used as an outlier score, indicating how anomalous a given case is. A high outlier score (reconstruction error) thus corresponds to a more anomalous case than a case with a low outlier score (reconstruction error). Henceforth the term outlier score will be used to refer to the model reconstruction error.

### Autoencoder: hyperparameter optimization

A larger grid search approach was used to train and evaluate multiple versions of autoencoder model hyperparameter settings across a grid of settings comprising neural architecture, learning rate, number of epochs and batch size (see supplemental Table 3) on the validation set. Default settings were used for the other hyperparameters to keep the search space reasonably bounded.

The optimized model (architecture; 128 × 64 × 64 × 128 neurons, 100 epochs, batch size = 64, learning rate; 0.01) achieved an AUROC of 0.886 on the validation set.

### Decision threshold

Once the model was optimized the validation set was used to set a decision threshold for the model, i.e. an outlier score threshold above which cases would be evaluated as anomalous and below which they would be evaluated as normal. So, for a given outlier score threshold, τ, and a given case, $y_{i}$, we set the prediction, $\widehat{{y_{i} }}$, as follows

$$\widehat{{y_{i} }} = \left\{ {\begin{array}{*{20}l} 1 \hfill & {if\;outlier\;score > \tau } \hfill \\ 0 \hfill & {if\;outlier\;score \le \tau } \hfill \\ \end{array} } \right.$$

where 1 = anomaly and 0 = normal. The aim was to find a threshold which maximized the number of cases in which normal patterns corresponded to non-intoxications and in which anomalous patterns corresponded to intoxication cases. All potential thresholds based on the ROC-curve of the validation set were tested to maximize the accuracy function

$${\mathrm{accuracy}}\left( {y,\hat{y}} \right) = \frac{1}{{n_{samples} }}\mathop \sum \limits_{i = 0}^{{n_{samples} - 1}} 1\left( {y_{i} = \widehat{{y_{i} }}} \right),$$

where $\widehat{{y_{i} }}$ is the predicted value (see above) of the i-th sample in the validation set and $y_{i}$ is the corresponding “true” value (intoxication = 1, non-intoxication = 0) and where 1(x) is the indicator function. Note that all samples were weighted equally, i.e. correct and incorrect classifications were weighted equally for intoxication and non-intoxication cases. Thus, an accuracy of 1.0 would indicate a complete correspondence between anomalous patterns and intoxications and between normal patterns and non-intoxications.

The highest accuracy of 0.856 (85.6%) on the validation set was achieved using a decision threshold of 10.02.

### Statistical methods and visualization tools

To investigate the correspondence between anomalous patterns and intoxication diagnoses cases the Kruskal–Wallis test was used to compare the number of detected substances between groups of cases in the test set (TP, FP, TN, FN). Dunn’s test was used for post-hoc evaluation^37^ adjusted with the Holm procedure for multiple comparisons^38^.

The Mann–Whitney U test was used when evaluating differences in outlier score between true positive and false positive cases (TP vs FP) and true negative and false negative cases (TN vs FN) respectively.

The Mann–Whitney U test was also used when comparing concentrations of substances between different groups together with corrections made for multiple comparisons using the Benjamini–Hochberg procedure^39^.

Uniform manifold approximation and projection (UMAP) was used in order to visualize the full high dimensional test set in two dimensions while preserving global structure^40^. When constructing the UMAP embedding concentration data were normalized by removing the mean and scaling to unit variance.

### Ethical considerations

All methods were carried out in accordance with relevant guidelines and regulations including the Declaration of Helsinki.

The study protocol was reviewed by the Swedish Ethical Review Authority (Etikprövningsmyndigheten), according to the board this retrospective registry-based study on deceased individuals does not fall within the scope of the Swedish Ethics Review Act and therefore did not require formal ethical approval. The authority issued an advisory opinion stating that it had no ethical objections to the study (reference number: 2023-06608-01).

Informed consent was waived due to the retrospective nature of the study and the use of data from deceased individuals.

## Results

### Population

The complete dataset (*n* = 16,710) included a majority of males (*n* = 12,288). The median age was 62 years, with a broad range (0–106 years, where 0 years denotes individuals younger than one year of age). Across all cases the dataset included 191 different substances and metabolites. Each included case had an average of 3.2 detected substances (median: 2, range 1–21). The median and mean number of detections per substance was 72 and 285 respectively across the whole dataset. See Table 1 for overall population characteristics. The most commonly found substances were ethanol (*n* = 6495), paracetamol (*n* = 2276) and zopiclone (*n* = 2221). For all list of included substances, and number of detections per substance, see supplemental Table 4).

**Table 1:** Population characteristics of the complete dataset (*n* = 16,710).

Sex	
Male *n* (%)	12,288 (73.5)
Female *n* (%)	4345 (26)
Not stated *n* (%)	77 (0.5)
Age	
Mean (SD)	59.94 (17.51)
Median (range)	62 (0–106)
Detected substances per case	
Mean (SD)	3.2 (2.5)
Median (range)	2 (1–21)
Number of detections per included substance	
Mean (SD)	285 (618)
Median (range)	72 (1–6495)

### Correspondence between anomalous patterns and intoxication diagnosis

**Fig. 2:**
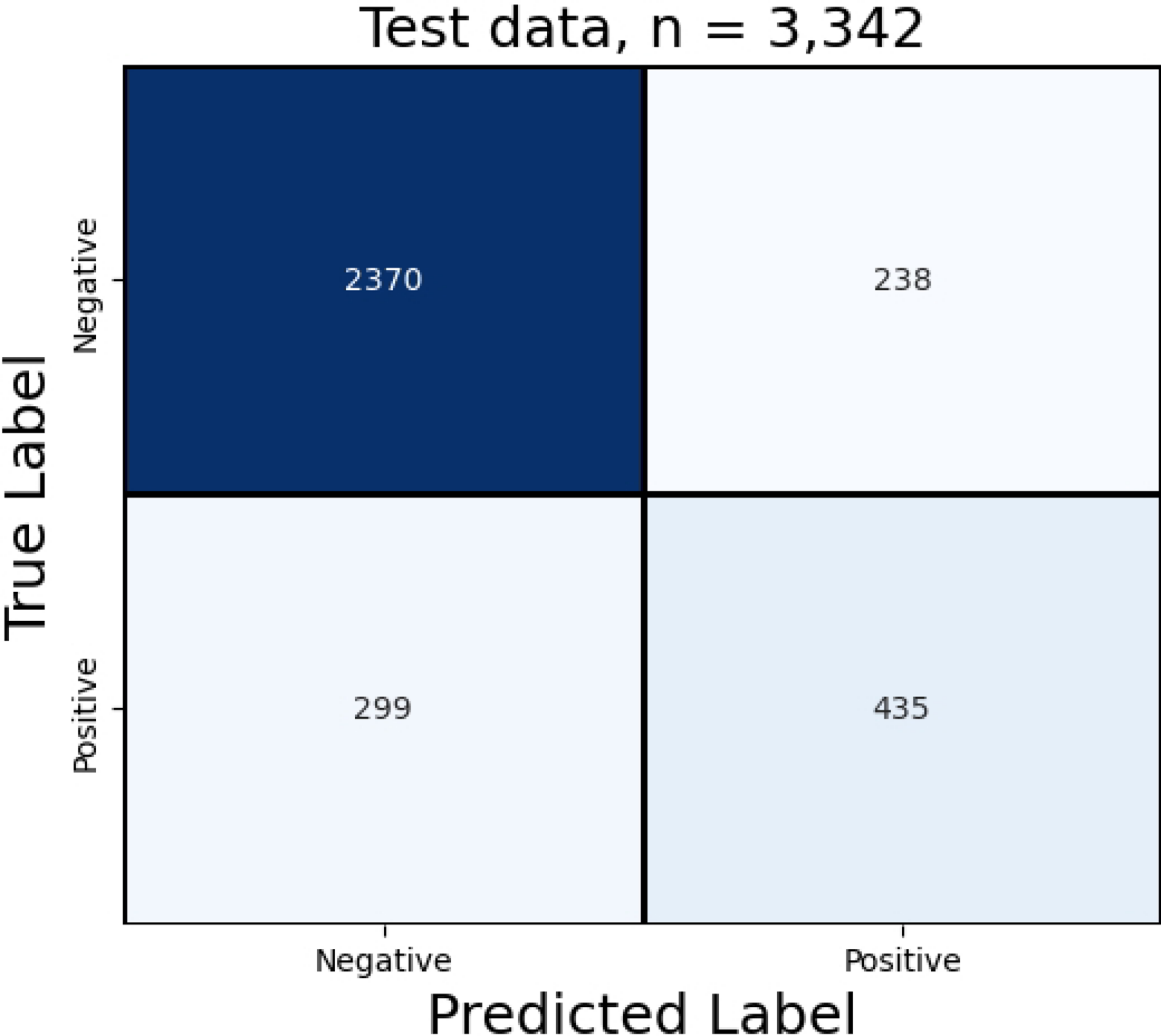
Confusion matrix. Confusion matrix of the test set.

The optimized autoencoder model achieved an AUROC of 0.879 on the test set (*n* = 3342). When applying the threshold derived from the validation set (10.2) the model had an overall accuracy of 83.9%, showing that, in general, anomalous and “normal” cases tend to correspond to intoxications and non-intoxications. However, the correspondence was higher for non-intoxications; with a negative predictive value (NPV) of 88.8%, while anomalous patterns had a positive predictive value (PPV) of 64.6%. The model had a sensitivity of 59.3% and specificity of 90.9%. False positives comprised 7.1% (*n* = 238) of the test set and false negatives made up 8.9% (*n* = 299). See Fig. 2 for the confusion matrix.

**Fig. 3:**
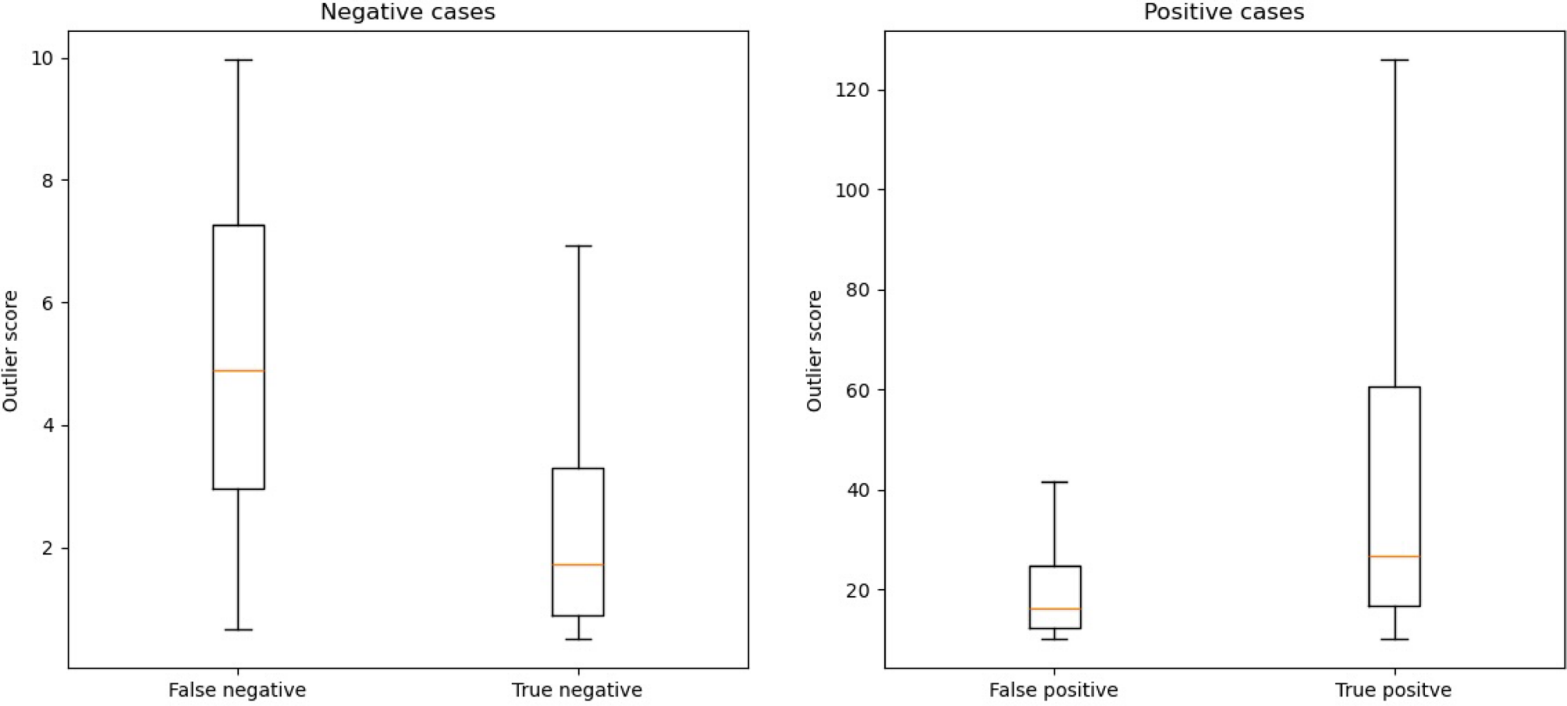
Distribution of outlier scores across classification groups. Threshold for model decision was an outlier score of 10.02. The box represents the interquartile range (IQR). Whiskers extend from the box to the furthest datapoint within IQR × 1.5. Outliers are not shown. Note the different scales on y-axis.

There were significant differences in outlier score between the true negative and false negative cases (*p* < 0.0001) and between true positive and false positive cases (*p* < 0.0001). In general, the outlier scores of the false negative cases (median = 4.89) and false positive cases (median = 16.15) were closer to the threshold (10.02) than the true negative cases (median = 1.72) and true positive cases (median = 26.6), see Fig. 3. Although, there is overlap between the two positive and two negative groups, the mismatched cases thus tend to be evaluated as less normal or less anomalous than the true negative and true positive groups.

### False positives: revealing potentially overlooked cases

False positives, cases with an anomalous toxicological pattern but without an intoxication diagnosis comprise 238 cases (7.1%) of the test set. Supplemental Table 5 shows the percentage of positive findings in each group of cases and Table 2, shows the top 25 substances more commonly found in false positive compared to true positive cases. Many of the substances more commonly found in false positive cases are not present among the true positive cases at all. Additionally, many of those substances are rare detections with 17 of 25 substances cases having ≤ 15 detections in the whole dataset.

**Table 2:** The top 25 substances more commonly found in false positive cases compared to true positive cases.

Substance	Relative frequency (false positive/true positive)
**Dabigatran**	*
Phenytoin	*
**Glibenclamide**	*
Carvedilol	*
**Memantine**	*
Mepivacaine	*
**Rivaroxaban**	*
Trimethoprim	*
**Bupvacaine**	*
**Dexamedetomidine**	*
**Edoxaban**	*
**Glipizide**	*
**Hydroxycarbazepine**	*
**Ketoprofen**	*
**Clobazam**	*
**Clonidine**	*
**Maprotiline**	*
**Norclobazolam**	*
**Pseudoephedrine**	*
**Reboxetine**	*
**Thioridazine**	*
Levetiracetam	16.5
Midazolam	16.4
Warfarin	14.6
Ketamine	11.9

Substances marked with a star (*) were only found in the false positive cases. Substances marked in bold are substances with few (≤ 15) detections in the complete dataset (*n* = 16,710).

Additionally, cases with higher number of detections tend to be evaluated as anomalous, see Table 4, with both false positive and true positive cases having higher number of detections (median 5 and 6 detections per case respectively) than true negative cases (median 2 detections per case) and the dataset as a whole (median 2 detections per case).

Finally, cases in the false positive group also tended to be composed of substances with higher concentrations than in the true negative group. For substances with positive detections in both groups (*n* = 125, i.e. disregarding substances for which there were only negative detections in either the false positive or true negative group) concentrations in the false positive group showed higher median concentrations for a majority of substances (*n* = 103). These higher concentrations constituted a significant (*p* < 0.05) difference for a subset of substances (*n* = 31), when correcting for multiple comparisons a smaller subset (*n* = 10) remained significant.

To illustrate the utility of identifying anomalous patterns a group of false positive cases and false negative cases were selected pseudorandomly to get a representation of outlier scores. Table 5 present these cases together with toxicological findings and the primary cause of death. The cases featuring higher outlier scores (403.7, 69.4 and 35.0 respectively) also feature substance concentrations which according to current postmortem reference concentrations correspond high and potentially toxic concentrations. The other two cases with lower outlier scores (20.5 and 15.1 respectively) feature a high number of detections, in which the combination of substances can lead to potentially harmful pharmacodynamic interactions.

### False negatives: limitations in identifying toxicological anomalies

As stated above 299 cases (8.9%) were false negative, i.e. they did not show anomalous toxicological patterns according to the model but had been diagnosed as intoxications by the attending forensic pathologist. However, as noted above, the negative predictive value (NPV) is overall high at 88.8%.

**Fig. 4:**
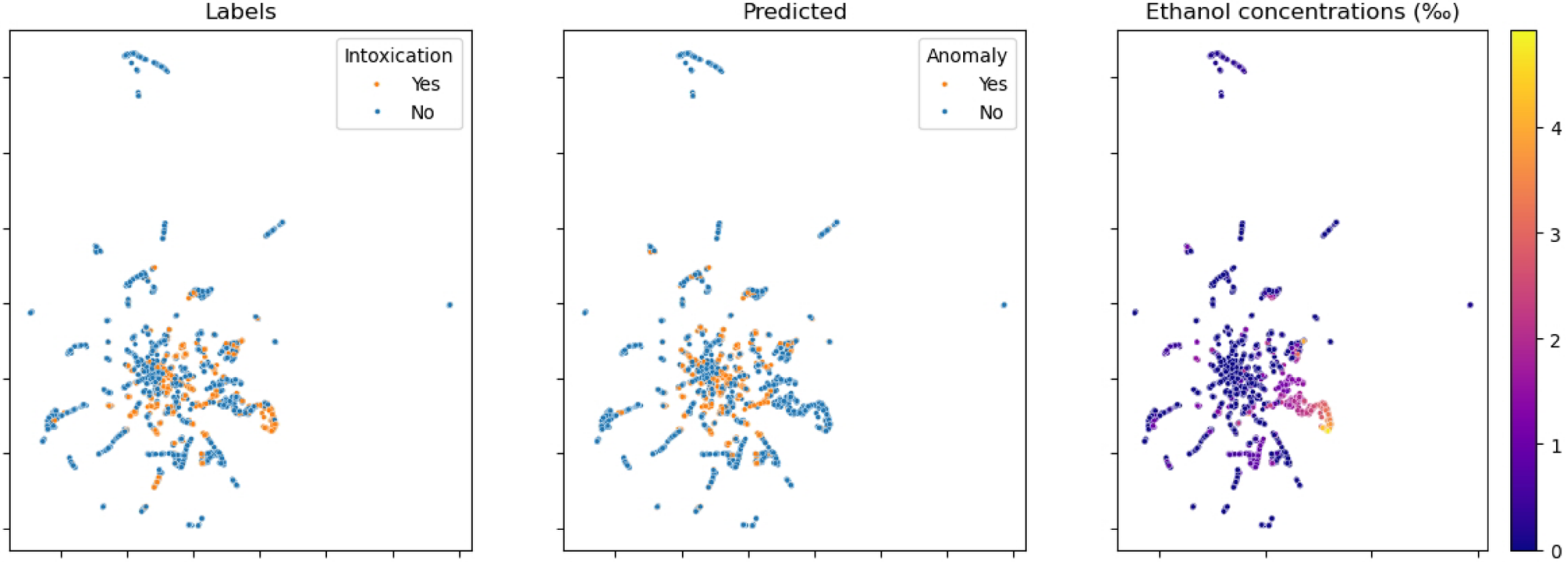
UMAP visualization. UMAP visualisation of the test set (*n* = 3342) showing the labelled data, model predictions and ethanol concentrations (colour coded according per mille concentration). Note the cluster of false negative cases with high ethanol concentrations.

Visualising the high dimensional test set in 2 dimensions using a UMAP projection, see Fig. 4, a large cluster of false negative cases (*n* = 74) in the middle right area becomes apparent. All these cases were found to contain ethanol with a concentration ≥ 2.94 ‰, indicating that the model tends to underestimate the degree to which high ethanol concentrations are anomalous.

**Fig. 5:**
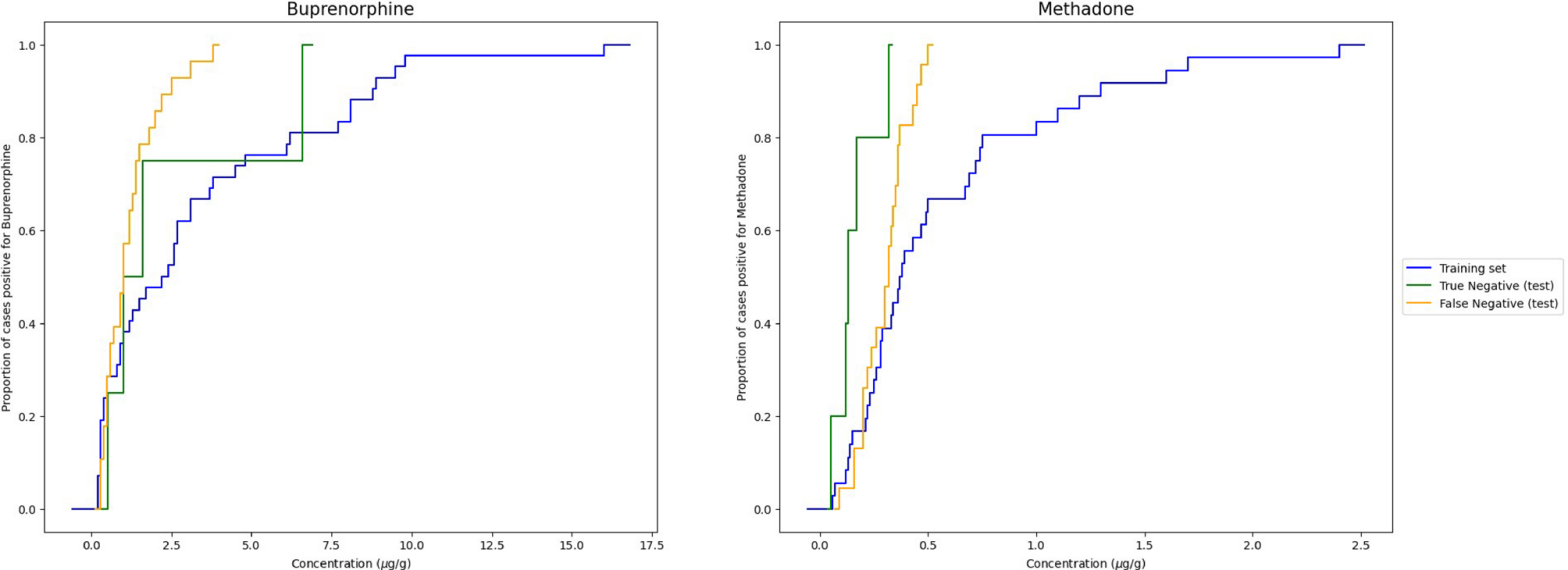
Buprenorphine and Methadone concentrations. Empirical cumulative distribution function graph (ECDF) showing the proportion of positive cases (concentration > 0 µg/g) for buprenorphine and methadone below a given concentration in the training set and test set. The training data is coloured blue, true negative test cases are green and false negative test cases are orange.

Generally, some additional broader trends can be observed. There are differences in the extent to which different substances are prevalent in true negative and false negative cases, supplemental Table 5 shows the percentage of positive findings in each group of cases. Table 3 shows the top 25 substances which were more frequently found in false negative compared to true negative cases. It can be noted that several opioids are more commonly present in the false negative cases. Dextropropoxyphene was not found in true negative cases at all. Additionally, 9.4% of all false negative cases contained buprenorphine and 7.7% contain methadone which is 55 and 37 times more common than in the true negative group respectively. These substances present a challenge due to a possible overlap between concentrations in intoxication and non-intoxication cases. Figure 5 show an empirical cumulative distribution function graph (ECDF) visualizing the proportion of cases positive for buprenorphine and methadone below a given concentration. The training set contains numerous cases with high concentrations of both buprenorphine and methadone, which in turn has informed the model’s knowledge on what constitutes a normal toxicological pattern.

**Table 3:** The top 25 substances more commonly found in false negative cases compared to true negative cases.

Substance	Relative frequency (false negative/true negative)
**Dextropropoxyphene**	*
EDDP	*
**Buprenorphine**	**55.5**
Norbuprenorphine	47.6
**Methadone**	**36.5**
Alprazolam	10.6
Clonazepam/7-Amino-Clonazepam	8.8
Levomepromazine	7.9
Desmethyllevomepromazine	7.9
**Hydrocodone**	**7.9**
Desmethylalimemzine	6.7
Pregabalin	6.6
Benzoylecgonine	6.34
Alimemazine	5.2
Methylphenidate	4.6
Ritalic Acid	4.3
**Codeine**	**4.1**
Cocaine	4.0
Amphetamine	3.9
Zolpidem	3.9
Nordazepam	3.8
**Morphine**	**3.6**
Diazepam	3.2
Quetiapine	3.2
Desmethylpromethazine	3.0

Substances marked with a star (*) were only found in the false negative cases and substances marked in bold are opioids.

In addition, there were significant differences in the number of detected substances per case between the different groups (*p* < 0.001), see Table 4. The true negative cases tend to have a lower number of detected substances (median 2 detections per case) compared to the false negative cases (median 3 detections per case). While there is a general trend that cases which the model identifies as anomalous have higher number of detections, the increased number of detections in the false negative group is not enough to inform the model of an anomalous pattern.

**Table 4:** Number of detected substances per case across groups.

Group	Median (range)	Mean (SD)
True positive	6 (1–21)	6.57 (3.11)
False positive	5 (1–14)	5.27 (2.82)
False negative	3 (1–14)	3.73 (2.38)
True negative	2 (1–10)	2.26 (1.60)

Table 5 present a pseudorandom selection of false negative cases (see above). Two cases, with outlier scores of 2.5 and 8.5 respectively, illustrate the difficulty of the model to assign an anomalous pattern to high concentrations of ethanol and opioids, in this case oxycodone. The case with the lowest outlier score of (0.7) feature pneumonia as the primary cause of death, potentially raising questions as to the impact of intoxication as a contributing cause. The two remaining cases, with outlier scores of 5.1 and 7.9, feature concentrations (amphetamine, pregabalin, zolpidem) in which there is some uncertainty in existing reference material if the measured concentrations should be considered toxic or not.

**Table 5:** A pseudo randomly selected set of 5 false positive and 5 false negative cases. Cases with an outlier score > 10.02 are false positives and cases with a score ≤ 10.02 are false negatives.

Sex (age)	Outlier score^a^	Toxicological findings (µg/g)	Primary cause of death	Comment
M (83)	403.7	Diazepam 28.0	Trauma	The concentration of diazpeam correspond to those seen in fatal intoxications^15^
		Metoprolol 0.07		
		Warfarin 0.42		
M (85)	69.4	Flukonazol 28.0	Urosepsis	Both concentrations are above the 90th and 95th percentiles respectively in a postmortem population^11^
		Trimetoprim 10.1		
M (61)	35.0	Aripirazol 0.44	Heart disease	The concentration of citalopram correspond to those seen in fatal intoxications^17^
		Dehydroaripiprazol 0.13		
		Citalopram 1.0		
		Desmethylcitalopram 0.21		
		Zuclopentixole 0.063		
F (39)	20.5	Amlodipine 0.04	Aortic aneurysm	The combination of aripiprazole, duloxetine, qutiapine, and propiomazine carries risk of sedation and anticholinergic effects^b^
		Aripiprazole 0.16		
		Duloxetine 0.44		
		Metoprolol 0.04		
		Quetiapine 0.78		
		Propiomazine 0.12		
M (32)	15.1	Alimemazine 0.2	Hanging	The combination of alprazolam, alimemazine, levomepromazine, olanzapine and zopiclone carries risk of sedation^b^ The combination of e.g. alimemazine, olanzapine and biperiden carries risk of anticholinergic effects. In addition substances such as citalopram and levomepromazine carries risk of QT-prolongation. Combining antidepressants also carries risk of sertonergic effects^b^ Olanzapine and levomepromazine carries risk of seizures^b^
		Desmethylalimemazine 0.2		
		Alprazolam 0.06		
		Biperiden 0.02		
		Citalopram 0.1		
		Levomepromazine 0.1		
		Desmethyllevomepromazine 0.2		
		Methylphenidate 0.005		
		Mirtazapine 0.1		
		Olanzapine 0.36		
		Desmethylolanzapine 0.13		
		Ritalic acid 0.22		
		Zopiclone 0.02		
F (63)	8.5	Oxycodone 0.76	Intoxication (oxycodone)	The concentration is above the 90th percentile in a postmortem population^7^. The anomaly detection model struggles with opioids, see Results and Discussion sections for further details
M (42)	7.9	Alprazolam 0.02	Intoxication (amphetamine, pregabaline)	The concentrations of alprazolam and morphine are low. The concentrations of amphetamine and pregabaline are below the 90th percentile in a postmortem population^7^
		Amphetamine 0.91		
		Morphine 0.013		
		Pregabaline 25		
F(42)	5.1	Alprazolam 0.02	Intoxication (ethanol, zolpidem, zopiclone)	The concentrations of alprazolam and zopiclone are low. The concentration of zolpidem corresponds both to those seen in non-intoxications and intoxications^15^
		Ethanol 0.93^c^		
		Zolpidem 0.31		
		Zopiclone 0.08		
M(57)	2.5	Ethanol 3.29^c^	Ethanol intoxication	The anomaly detection model struggles with ethanol; see Results and Discussion sections for further details
M(37)	0.7	Ethanol 0.97^c^	Pneumonia (intoxication as contributing cause)	The concentration of morphine is low^11^. The primary cause of death is not an intoxication
		Morphine 0.01		

^a^A measure of how anomalous a given case is. High values indicate a more anomalous case. See methods section on reconstruction error and outlier score for further details. The model classifies cases with an outlier score above 10.02 as anomalous.

^b^Information regarding potentially harmful interactions are gathered from a Swedish interaction database (www.janusinfo.se).

^c^Concentration in per mille (‰).

## Discussion

Current postmortem reference concentrations in forensic toxicology struggle to evaluate cases involving multiple substances. When interpreting multiple concentrations there is currently no tool to separate common patterns seen in non-intoxication cases from uncommon patterns, potentially indicating intoxications or other cases meriting further review. One potential approach to identifying uncommon patterns in high dimensional data is to apply anomaly detection methods. This work presents an anomaly detection autoencoder model as a potential evaluation tool in postmortem toxicology. The model was trained on a large dataset of postmortem non-intoxication cases (*n* = 8320) and is capable of simultaneously evaluating complete toxicological profiles. This enables an integrated assessment of multiple drugs and pharmaceuticals acting in combination.

The model demonstrates a high specificity for identifying normal and non-intoxication cases while taking all detected substances into account. Alignment with diagnoses of fatal intoxication is lower, reflecting the more complex relationship between anomalous toxicological patterns and intoxication. The observed sensitivity reflects the correspondence between the anomalous toxicological patterns identified by the model and the current forensic diagnosis of fatal intoxication. Cases classified as false positives may nevertheless exhibit toxicological profiles of forensic interest. Consequently, sensitivity with respect to intoxication should be interpreted in the context of the model’s intended purpose, to identify anomalous toxicological patterns that may warrant further forensic evaluation.

### Selection of a validation set: anomalies or intoxications

Evaluating and optimizing the performance of an anomaly detection model is a complex task. In anomaly detection in general, it is often unclear from the outset what constitutes an anomalous pattern as this is normally not defined in the dataset itself. Therefore, when training anomaly detection models, default model settings (hyperparameters) or other preset recommended settings are commonly used, i.e. the hyperparameters are not tailored specifically for the dataset onto which the model is applied. However, current research indicates that it is preferable to evaluate and optimize the model’s hyperparameters on a validation set with labelled data rather than using default settings^41,42^. In the present study, our validation set does not have labelled anomalous data as it is unclear how to best define an anomaly in this context. We instead proceeded with the assumption that intoxication cases contain a larger proportion of anomalous patterns than non-intoxications. Thus, for evaluation and optimization of models, we considered the current labels on the validation set (intoxication and non-intoxication) as reasonable substitutes for anomalous and normal patterns, even if full correspondence cannot be assumed.

The use of intoxication diagnoses as surrogate labels has implications for the interpretation of model performance. It should be noted that the diagnosis of intoxication is not entirely independent of the toxicological data used as model input, as toxicological findings contribute to the forensic assessment of the cause of death. Consequently, the selected decision threshold and the reported performance metrics are, to some extent, influenced by current forensic toxicological practice and the interpretation of postmortem drug concentrations. However, because the model was trained exclusively on non-intoxication cases, this dependence primarily affects the evaluation of model performance rather than the learning of normal toxicological patterns.

### Potential impact of population factors

The current model does not perform a direct pharmacodynamical analysis of the findings. Instead, the model’s decision is based on the content of the training set. Thus, what the model evaluates as normal or anomalous is dependent of what is commonly found in the training set. This is in turn based on the makeup of the population that undergoes a forensic autopsy in Sweden as well as national prescription and drug abuse patterns. Cases eligible for a forensic autopsy in Sweden include all cases of suspected or obvious unnatural death, deaths involving unclear circumstances and cases in which the identity of the deceased is unknown.

Data from the Swedish Medical Products Agency regarding prescription medication 2006–2023^43^ show that the drug classes with the highest prevalence in the general population are antibiotics, non-opioid painkillers and antihypertensive agents. More recently, data from 2023 show that antidepressants as the most common prescribed drug among females aged 18–64, and the second most prescribed drug for males in the same age group. According to the Swedish National Board of Health and Welfare the most common substances in fatal intoxications are heroin, oxycodone and alimemazine. In suicidal intoxications the most common substances are instead zopiclone, propiomazine and oxycodone^44^. Among the Nordic countries intoxications among substance abusers tend to mainly involve opioids^20^.

Thus, in general, the pattern of detections which the current model evaluates as anomalous could change with the inclusion of additional data or data from regions which present a different drug panorama. Of special note is that pharmaceutical intoxications are common in the western world^45^, in other parts of the world intoxications with e.g. pesticides are more common^46^. To provide the best results the model should be trained on representative data from the population in which it is to be applied.

### Case selection considerations

In the present study we wanted to explore the performance of an anomaly detection approach under stringent and rigorous conditions to aid the analysis of the results. We therefore only included cases that had been subject to the same analytical pipeline. This was done to avoid a potential confounding factor to the model analysis. The analytical strategy might be different in cases of high suspicion of intoxication involving a broader or more targeted spectrum of analysis than in cases with low suspicion of intoxication. To that end the present model does not consider the presence of e.g. designer drugs, atypical benzodiazepines or new synthetic opioids. Adopting broader inclusion criteria could potentially increase the number of intoxication cases with the presence of rare substances, which could result in a lower frequency of false positive cases. Additionally, it could provide a larger pool of cases for the training set providing more data for characterizing anomalous cases. However, using such an approach could introduce difficulty in interpreting if a pattern is anomalous because of the findings in themselves or because of the analytical strategy employed in the case.

### Comparison to current reference concentrations

To our knowledge there is currently no available reference concentrations which evaluate and adapt to a complete toxicological profile simultaneously. Current high-quality collections of postmortem reference concentrations, such as those based on Finish data by Ketola et al.^11^ and the Swedish series^13–17^, focus on single substance reference concentrations making direct comparisons with the current model difficult. However, an indirect comparison can be made by supplying the model with increasing concentrations of single substance alone (i.e. all other concentrations are set to zero) and noting at which concentration threshold the model indicates an anomaly based on the presence of that single substance alone. In Table 6 a selection of single substances alone is tested by the current model and the concentration threshold in which each substance is designated an anomaly is compared with current reference concentrations by Ketola et al. (containing all postmortem cases in which a substance has been detected) and the series of studies using the Swedish-method (postmortem cases separated into a control group and two intoxication groups). While it must be noted that this scenario, i.e. investigating only a single substance, does not necessarily correspond to how the model was trained the concentration thresholds do correspond well with either high concentration in the Ketola study or single substance intoxication concentrations in the studies using the Swedish studies or both, with the exception of amitriptyline and levomepromazine which are anomalous at lower concentrations.

**Table 6:** Anomaly threshold for a selection of substances compared with corresponding concentration thresholds from postmortem reference compilations.

Substance	Detections in dataset^a^ (n)	Anomaly threshold^b^ (μg/g)	Ketola et al.^11^		Reis et al.^14^		Jönsson et al.^15^		Söderberg et al.^16^	
			90th percentile (μg/g) of all cases	95th percentile (μg/g) of all cases	90th percentile of control cases	10th percentile of single substance intoxications	90th percentile of control cases	10th percentile of single substance intoxications	90th percentile of control cases	10th percentile of single substance intoxications
Amitriptyline	305	0.51	2.6	4.6	0.4	1.0				
Citalopram	1317	1.78	1.3	2.1	0.7	1.5				
Mirtazapine	1736	0.89	0.7	1.5	0.3	1.0				
Alprazolam	921	0.18	0.12	0.2			0.12	0.13		
Diazepam	1223	0.70	0.4	0.6			0.4	1.2		
Oxazepam	949	1.46	0.69	1.2			0.9	2.0		
Clozapine	102	1.39	4.3	8.7					1.55	1.24
Levomepromazine	175	0.23	1.9	3.2					0.4	0.8
Olanzapine	572	0.35	0.8	1.4					0.2	0.29

^a^Total number of detections across the whole dataset (*n* = 16,710).

^b^The concentration at which the model designates the toxicological profile of this substance evaluated in isolation as anomalous.

For a more realistic example we need to consider multiple substances simultaneously, see Table 7, as adding the concurrent presence of other substances and metabolites will modify the anomaly threshold. We in this case use citalopram as an example. Allowing for the presence of the major metabolite to citalopram, desmethylcitalopram, at half the concentration of the parent substance lowers the concentration at which citalopram is evaluated as anomalous to 1.38 µg/g which also correspond well to current reference concentrations in a single substance intoxication scenario (10th percentile of single substance intoxications 1.09–1.5 µg/g)^14,17^. Adding the presence of another substance to the scenario will again modify the concentration at which citalopram is deemed anomalous. Adding 0.6 µg/g alimemazine, a concentration seen in multi-substance intoxications according to Söderberg et al.^16^, lowers the concentrations at which citalopram is deemed anomalous to 0.72 µg/g corresponding to the concentrations seen in multi-substance intoxications involving citalopram (10th percentile of multiple substance intoxications 0.7 µg/g)^14,17^.

**Table 7:** Anomaly threshold for citalopram with co-detections compared with corresponding concentration thresholds from postmortem reference compilations.

Substance	Anomaly threshold^b^ (μg/g)	Reis et al.^14^		Söderberg et al.^17^	
		10th percentile of single substance intoxications of citalopram (μg/g)	10th percentile of multisubstance intoxications involving citalopram (μg/g)	10th percentile of single substance intoxications of citalopram (μg/g)	10th percentile of multisubstance intoxications involving citalopram (μg/g)
Citalopram (alone)	1.78	1.5		1.09	
Citalopram + desmethylcitalopram^a^	1.38	1.5		1.09	
Citalopram + desmethylcitalopram^a^ + 0.6 μg/g alimemazine	0.72		0.7		0.7

^a^Desmethylcitalopram set to half the concentration of citalopram.

^b^The concentration at which the model designates the toxicological profile of citalopram as anomalous.

As stated above, the isolated evaluation of a single substance, without other concurrent findings or presence of metabolites, is an out-of-context scenario for the current model. While the discussion above indicates that the anomaly threshold in single substance scenarios can often provide useful and relevant information, the models judgement in these cases should be supplemented with a manual evaluation based on single substance reference information.

### False negatives and false positives: pitfalls and opportunities

Neural network models are often regarded as “black boxes” because, although the input data and the resulting predictions are observable, the internal representations and computations that transform the inputs into predictions are not readily interpretable. Consequently, it is difficult to determine how individual input features contribute to the model’s predictions. Nevertheless, some trends can be identified from the evaluation of the test set.

Regarding false negatives, the model tends to underestimate the extent to which ethanol can contribute to an anomalous pattern, as even high ethanol concentrations are not evaluated as anomalous. The threshold at which the model evaluates a case containing only ethanol as anomalous is 10.89 ‰, which is much higher than what is normally considered potentially fatal in postmortem cases^47^. One possible reason for this is the frequency ethanol is found in the full dataset (*n* = 6495). As ethanol is the most commonly found substance the model will have been extensively exposed to ethanol concentrations in training thus potentially minimizing the autoencoder reconstruction error (the outlier score) when ethanol is encountered resulting high concentrations of ethanol needed to surpass the anomaly threshold. This could be considered a limitation of the anomaly detection approach to toxicological pattern evaluation; very common substances can be underestimated in their contribution to anomalous patterns.

The other trend in false negative cases concerns opioids. The toxicological pattern in these cases does not seem to strongly correspond to whether cases are considered intoxications or not. Using buprenorphine as an example, see Fig. 5, the training set contains a broad range of concentrations, potentially causing the model to evaluate those same concentrations as normal when applied to the test set. It is well known that tolerance is an important factor in evaluating the effect of opioids, with a large overlap between concentrations judged as normal and toxic^48^. The current model does not take tolerance into account. Evaluation of opioid concentrations in isolation is therefore not enough to correctly diagnose these cases. Additional tools of evaluation need to be employed to increase diagnostic accuracy, such as hair testing^49^ or autopsy findings^50^.

False positives may be viewed as opportunities to identify cases of potential forensic interest that are not detected by existing reference concentrations. These cases are typically characterized by the presence of rare substances, a high number of detected compounds, and elevated concentrations. Because such features are anomalous relative to both the full dataset and the training set, this model behaviour is expected. Importantly, the model is designed to identify deviations from the norm, and classification as anomalous does not necessarily imply evidence of intoxication. Nevertheless, the distinctive characteristics of these false positive cases make them valuable candidates for targeted review, as illustrated by the examples in Table 5, although the forensic significance of these anomalies requires case-specific evaluation and remains to be established.

In summary, the correspondence between anomalous toxicological patterns and intoxications is lower in cases including very common substances, in this case ethanol, and where the concentration of a substance is an insufficient tool for a correct evaluation, as with opioids. However, the model correctly evaluates cases with rare substances, high number of detections or high concentrations as anomalous, and while these characteristics do not necessarily correspond to fatal intoxication or other toxicologically relevant findings, they may identify cases that warrant further review.

## Limitations

While the present study is focused on the identification and use of anomalous toxicological patterns as a screening tool, it must be acknowledged that postmortem toxicological interpretation also requires careful consideration of case circumstances, autopsy findings, and other case-specific factors. The current model does not incorporate these elements. Alternative frameworks, such as the Toxicological Significance Score (TSS)^51,52^, aim to integrate toxicological findings with pathological and circumstantial information when assessing the contribution of detected substances to death. The proposed model is intended to complement such comprehensive forensic evaluations. Thus, while the evaluation of a toxicological pattern may provide a useful screening tool, it should be applied alongside established forensic assessment methods in comprehensive casework.

Additionally, as discussed above, the model results are influenced by the available training set and the characteristics thereof. Increasing the size of the training set or including cases from a more diverse pool of cases could increase the accuracy and applicability of the model assessments.

Substances that were not detected were represented by zero concentrations, allowing the model to treat non-detected analytes as part of the overall toxicological profile. Future work could investigate whether alternative encoding strategies improve model performance.

We also acknowledge that, for certain substances, there is limited correspondence between anomalous patterns and intoxication diagnoses (e.g., opioids and ethanol). For ethanol, this is likely related to its high prevalence in the training data, whereas for opioids, substantial overlap between therapeutic and toxic concentrations and the influence of tolerance reduce the correspondence between toxicological patterns and intoxication diagnoses. These limitations should be considered when interpreting the model output.

While false positive cases offer opportunities to identify cases of forensic interest, future efforts should also focus on addressing false negatives. To improve sensitivity to intoxication cases, the current anomaly detection model could be complemented by classification models trained to distinguish between intoxication and non-intoxication cases, rather than to detect anomalous patterns. Combining these approaches in an ensemble framework may enable more nuanced and robust case assessment.

## Conclusions

The current study provides a proof of concept for a data-driven approach to identifying toxicological patterns of relevance and serves as a first step towards comprehensively evaluating postmortem polydrug findings. While the model seems to provide reasonable results even when presented with single concentrations, further work is needed to increase sensitivity and accuracy in identifying cases currently judged as intoxications. The high accuracy in identifying normal cases and the ability to identify anomalous patterns in general indicate that the model has potential as a decision-support screening tool to prioritize cases for further review by the forensic toxicologist. The model is intended to complement established forensic assessment methods.

## Supplementary Information

Below is the link to the electronic supplementary material.

Supplementary Material 1

## Data Availability

The data that support the findings of this study are available from the corresponding author upon reasonable request. Due to legal and ethical considerations, the data is not publicly available.
