# Supplementary material for: Anomaly detection as a tool for identifying cases of interest in postmortem toxicology
Source: Sci Rep. 2026 Aug 17;16:25686. doi: 10.1038/s41598-026-65664-5 (PMC13482327; doi:10.1038/s41598-026-65664-5)
Supplement: Supplementary file 1 [file 41598_2026_65664_MOESM1_ESM.docx]

Supplemental Table 1

Excluded causes of death

| **Intoxications** | | |
| --- | --- | --- |
| **Cause of death** | **Modified ICD-9 code^1^** | **ICD-10 code^2^** |
| Intoxications with hormones and synthetic substitutes | 962D |  |
| Intoxication with insulin and antidiabetic substances | 962 | T38.3, T383 |
| Toxic effect of carbon monoxide | 986 |  |
| Carbon monoxide poisoning (motor exhaust) | 986K |  |
| Carbon monoxide poisoning (fire) | 986M | T58 |
| Toxic effect of condensed petroleum gases | 987A | X4790 |
| Toxic effect of hydrogen cyanide gas | 987H |  |
| Toxic effect of carbon monoxide and cyanide |  | T589 |
| Toxic effect of specific gases (other than 9874A-H) | 987W |  |
| Toxic effect of gases, unspecified | 987X |  |
| Toxic effect of mushrooms | 988B | T620 |
| Toxic effect of GHB | 989W |  |
|  |  |  |
| **Unspecific but substance related codes** | | |
| **Cause of death** | **Modified ICD-9 code^1^** | **ICD-10 code^2^** |
| Chronic alcoholism | 303 | F10.2 |
| Substance abuse syndrome | 304, 305 |  |
| Alcoholic dependence syndrome | 303X | F102A, F102X |
| Alcohol related dementia |  | F107 |
| Drug addiction, morphine and other opiods | 304A |  |
| Drug addiction, amphetamine and other stimulants | 304E |  |
| Drug addiction, combination of different drugs | 304H |  |
| Drug addiction, specific drugs other than 304A-H | 304W |  |
| Substance abuse, mixed | 304X, 305X | F111, F119, F131, F15, F19, F193, F99, F999, Z722, Z814, Z864 |
| Alcohol abuse | 305A | Y919 |
| Side effect of pharmaceutical substances | 995C | F11, T887, T969, Y450, Y475, Y490, Y529, Y578, Y579, Y880 |
| Psychiatric effects secondary to substance abuse |  | F100, F101, F102, F110, F112, F129, F130, F14, F141, F151, F152, F190, F191, F192, F199 |
| Ingestion of cocaine |  | T405 |

1 The National Board of Forensic Medicine in Sweden uses a modifed version of the ICD-9 coding system with added prefixes and suffixes

2 During a short period in 2012 ICD-10 codes was used instead of the modified ICD-9 system

Supplemental Table 2

Initial model selection based of different methodologies

| **Model** | **Neural network structure** | **AUROC^1^** |
| --- | --- | --- |
| Autoencoder^2^ | [128, 64, 32]^3^ | 0.873 |
|  | [128, 64] ^3^ | 0.875 |
|  | [64, 32] ^3^ | 0.875 |
|  |  |  |
| Deep Isolation Forest | [128, 64, 32] | 0.740 |
|  | [128, 64] | 0.819 |
|  | [64, 32] | 0.819 |
|  |  |  |
| Deep One-Class Classification (DeepSVDD)^2^ | [128, 64, 32] | 0.871 |
|  | [128, 64] | 0.872 |
|  | [64, 32] | 0.854 |
|  |  |  |
|  | Maximum samples^4^ |  |
| Isolation Forest^5^ | 128 | 0.837 |
|  | 256 | 0.833 |
|  | 512 | 0.861 |

1 AUROC; Area under the receiver operating characteristic curve. Rounded to three decimal spaces.

2 Number of epochs set to 50 for these models

3 Architecture is mirrored, e.g. [64,32] indicates [64,32,32,64]

4 Number of samples used to build each base estimator

5 A tree-based anomaly detection model which does not use neural networks

Supplemental Table 3

Performance of autoencoder model on validation set (AUROC) trained using different hyperparameter settings. AUROC values are rounded to three decimal spaces.

| **Architecture** | **Learning rate** | **Epochs** | **Batchsize = 16** | **Batchsize = 32** | **Batchsize = 64** |
| --- | --- | --- | --- | --- | --- |
| [128, 64, 32] | 0.001 | 10 | 0.871 | 0.868 | 0.865 |
| [128, 64, 32] | 0.001 | 20 | 0.871 | 0.873 | 0.868 |
| [128, 64, 32] | 0.001 | 50 | 0.871 | 0.873 | 0.873 |
| [128, 64, 32] | 0.001 | 100 | 0.872 | 0.870 | 0.873 |
| [128, 64, 32] | 0.01 | 10 | 0.861 | 0.862 | 0.868 |
| [128, 64, 32] | 0.01 | 20 | 0.864 | 0.869 | 0.868 |
| [128, 64, 32] | 0.01 | 50 | 0.862 | 0.867 | 0.868 |
| [128, 64, 32] | 0.01 | 100 | 0.865 | 0.869 | 0.873 |
| [128, 64, 32] | 0.1 | 10 | 0.876 | 0.877 | 0.877 |
| [128, 64, 32] | 0.1 | 20 | 0.877 | 0.878 | 0.877 |
| [128, 64, 32] | 0.1 | 50 | 0.876 | 0.876 | 0.876 |
| [128, 64, 32] | 0.1 | 100 | 0.877 | 0.877 | 0.877 |
| [128, 64, 32] | 1 | 10 | 0.874 | 0.874 | 0.877 |
| [128, 64, 32] | 1 | 20 | 0.878 | 0.879 | 0.877 |
| [128, 64, 32] | 1 | 50 | 0.876 | 0.875 | 0.886 |
| [128, 64, 32] | 1 | 100 | 0.879 | 0.877 | 0.877 |
| [128, 64] | 0.001 | 10 | 0.872 | 0.871 | 0.871 |
| [128, 64] | 0.001 | 20 | 0.871 | 0.872 | 0.871 |
| [128, 64] | 0.001 | 50 | 0.864 | 0.875 | 0.869 |
| [128, 64] | 0.001 | 100 | 0.868 | 0.881 | 0.873 |
| [128, 64] | 0.01 | 10 | 0.871 | 0.873 | 0.872 |
| [128, 64] | 0.01 | 20 | 0.877 | 0.875 | 0.870 |
| [128, 64] | 0.01 | 50 | 0.872 | 0.875 | 0.879 |
| [128, 64] | 0.01 | 100 | 0.866 | 0.873 | 0.886 |
| [128, 64] | 0.1 | 10 | 0.876 | 0.878 | 0.877 |
| [128, 64] | 0.1 | 20 | 0.878 | 0.877 | 0.877 |
| [128, 64] | 0.1 | 50 | 0.878 | 0.878 | 0.877 |
| [128, 64] | 0.1 | 100 | 0.877 | 0.877 | 0.877 |
| [128, 64] | 1 | 10 | 0.880 | 0.873 | 0.868 |
| [128, 64] | 1 | 20 | 0.877 | 0.878 | 0.877 |
| [128, 64] | 1 | 50 | 0.863 | 0.876 | 0.878 |
| [128, 64] | 1 | 100 | 0.883 | 0.877 | 0.877 |
| [64, 32] | 0.001 | 10 | 0.871 | 0.870 | 0.867 |
| [64, 32] | 0.001 | 20 | 0.867 | 0.873 | 0.869 |
| [64, 32] | 0.001 | 50 | 0.868 | 0.875 | 0.875 |
| [64, 32] | 0.001 | 100 | 0.871 | 0.873 | 0.878 |
| [64, 32] | 0.01 | 10 | 0.862 | 0.867 | 0.868 |
| [64, 32] | 0.01 | 20 | 0.871 | 0.868 | 0.871 |
| [64, 32] | 0.01 | 50 | 0.871 | 0.875 | 0.879 |
| [64, 32] | 0.01 | 100 | 0.875 | 0.879 | 0.875 |
| [64, 32] | 0.1 | 10 | 0.877 | 0.876 | 0.876 |
| [64, 32] | 0.1 | 20 | 0.877 | 0.877 | 0.876 |
| [64, 32] | 0.1 | 50 | 0.878 | 0.877 | 0.878 |
| [64, 32] | 0.1 | 100 | 0.878 | 0.878 | 0.878 |
| [64, 32] | 1 | 10 | 0.877 | 0.875 | 0.875 |
| [64, 32] | 1 | 20 | 0.877 | 0.877 | 0.878 |
| [64, 32] | 1 | 50 | 0.877 | 0.879 | 0.876 |
| [64, 32] | 1 | 100 | 0.878 | 0.878 | 0.879 |

1 Architecture is mirrored, e.g. [64,32] indicates [64,32,32,64]

Supplemental Table 4

Included substances and number of detections in the complete dataset (n = 16,710)

| **Substance** | **Detections** |
| --- | --- |
| 6-acetylmorphine | 118 |
| 7-hydroxiquetiapin | 79 |
| Alimemazine | 956 |
| Alprazolam | 921 |
| Amiodarone | 580 |
| Amitriptyline | 305 |
| Amlodipine | 1397 |
| Amphetamine | 442 |
| Apixaban | 75 |
| Aripiprazole | 118 |
| Atenolol | 539 |
| Atomoxetine | 14 |
| Atropine | 47 |
| Baclofen | 39 |
| Benzoylecgonine | 147 |
| Biperiden | 108 |
| Bisoprolol | 635 |
| Bromazepam | 12 |
| Bupivacaine | 6 |
| Buprenorphine | 330 |
| Bupropion | 216 |
| Buspirone | 75 |
| Caffeine | 120 |
| Carbamazepine | 302 |
| Carisoprodol | 1 |
| Carvedilol | 16 |
| Chlorfeniramine | 7 |
| Chloroquine | 12 |
| Chlorprothixene | 16 |
| Citalopram | 1317 |
| Clobazam | 3 |
| Clometihazole | 17 |
| Clomipramine | 178 |
| Clonazepam /  7-amino-clonazepam | 471 |
| Clonidine | 11 |
| Clozapine | 102 |
| Cocaine | 76 |
| Codeine | 820 |
| Dabigatran | 10 |
| Dehydroaripiprazole | 49 |
| Desmethylalimemazine | 626 |
| Desmethylcitalopram | 925 |
| Desmethylclomipramine | 161 |
| Desmethyllevomepromazine | 97 |
| Desmethylmianserine | 42 |
| Desmethylmirtazapine | 1539 |
| Desmethylolanzapine | 292 |
| Desmethylprometazine | 313 |
| Desmethylsertraline | 774 |
| Desmetyhlclozapine | 94 |
| Dexmedetomidine | 6 |
| Dextromethorphan | 16 |
| Dextropropoxyphene | 42 |
| Dextrorphan | 1 |
| Diazepam | 1223 |
| Diclofenac | 31 |
| Dihydrocodeine | 5 |
| Diltiazem | 31 |
| Diphenhydramine | 11 |
| Doxylamine | 7 |
| Duloxetine | 355 |
| EDDP | 108 |
| Edoxaban | 3 |
| Ephedrine | 144 |
| Ethanol | 6495 |
| Ethylmorphine | 141 |
| Ethylphenidate | 34 |
| Etoricoxib | 72 |
| Felodipine | 7 |
| Fentanyl | 374 |
| Flecainide | 21 |
| Fluconazole | 53 |
| Flunitrazepam /  7-amino-flunitrazepam | 166 |
| Fluoxetine | 254 |
| Flupentixol | 55 |
| Fluphenazine | 3 |
| Fluvoxamine | 3 |
| Gabapentin | 353 |
| Glibenclamide | 9 |
| Glipizide | 5 |
| Haloperidol | 182 |
| Hydrocodone | 68 |
| Hydromorphone | 10 |
| Hydroxichloroquine | 11 |
| Hydroxirisperidone | 279 |
| Hydroxizine | 658 |
| Hydroxybupropion | 197 |
| Hydroxycarbazepine | 9 |
| Isopropanol | 82 |
| Ketamine | 191 |
| Ketobemidone | 47 |
| Ketoprofen | 3 |
| Labetalol | 42 |
| Lacosamide | 11 |
| Lamotrigine | 358 |
| Lercanidipine | 2 |
| Levetiracetam | 110 |
| Levomepromazine | 175 |
| Lidocaine | 153 |
| Loperamide | 41 |
| Lorazepam | 29 |
| Maprotiline | 2 |
| MDA | 18 |
| MDMA | 28 |
| Melperone | 9 |
| Memantine | 15 |
| Mepivacaine | 23 |
| Meprobamat | 1 |
| Metformin | 213 |
| Methadone | 329 |
| Methamphetamine | 23 |
| Methanol | 6 |
| Methylphenidate | 245 |
| Methylscopolamine | 1 |
| Metoclopramide | 149 |
| Metoprolol | 1991 |
| Metronidazole | 18 |
| Mianserin | 61 |
| Midazolam | 147 |
| Mirtazapine | 1736 |
| Moclobemide | 11 |
| Morphine | 1194 |
| N-desethylamiodarone | 274 |
| Naproxene | 187 |
| Nifedipine | 1 |
| Nitrazepam /  7-amino-nitrazpam | 279 |
| Norbuprenorphine | 275 |
| Norclobazam | 3 |
| Nordazepam | 1292 |
| Norephedrine | 30 |
| Norfluoxetine | 271 |
| Nortriptyline | 243 |
| Norverapamil | 29 |
| Noscapine | 10 |
| O-desmethylvenlafaxine | 632 |
| O-desmetyhltramadol | 497 |
| Olanzapine | 572 |
| Orphenadrine | 19 |
| Oxazepam | 949 |
| Oxikodone | 926 |
| Papaverine | 1 |
| Paracetamol | 2276 |
| Paroxetine | 186 |
| Perphenazine | 84 |
| Pethidine | 4 |
| Phenazepam | 1 |
| Phenazone | 8 |
| Phenytoin | 25 |
| Pindolol | 2 |
| Pregabalin | 713 |
| Primidone | 3 |
| Prometazine | 489 |
| Propranolol | 210 |
| Prropiomazine /  dihydropropiomazine | 1400 |
| Pseudoephedrine | 6 |
| Quetiapine | 325 |
| Quinine | 23 |
| Reboxetine | 7 |
| Reduced haloperidol | 201 |
| Risperidone | 142 |
| Ritalinic acid | 258 |
| Rivaroxaban | 13 |
| Ropivacaine | 14 |
| S-metyldihydroziprasidone | 10 |
| Sertraline | 905 |
| Sotalol | 47 |
| Tamoxifen | 20 |
| Tapentadol | 15 |
| Temazepam | 131 |
| Teopylline | 8 |
| Thioridazine | 1 |
| Topiramate | 43 |
| Tramadol | 583 |
| Triazolam | 2 |
| Trihexyphenidyl/benzhexol | 25 |
| Trimethoprim | 38 |
| Trimipramine | 1 |
| Venlafaxine | 649 |
| Verapamil | 36 |
| Vortioxetine | 66 |
| Warfarin | 272 |
| Zaleplon | 1 |
| Ziprasidone | 15 |
| Zolpidem | 611 |
| Zonisamide | 3 |
| Zopiclone | 2221 |
| Zuklopenthixol | 172 |

Supplemental Table 5

Percentage of positive detections (positive detections / all detections in the group) of substances in the test set (n = 3,342) across true positive, false positive, true negative and false negative groups. Values are rounded to three decimal places.

| **Substance** | **True positive** | **False positive** | **True negative** | **False negative** |
| --- | --- | --- | --- | --- |
| 6-acetylmorphine | 0.041 | 0.000 | 0.000 | 0.000 |
| 7-hydroxiquetiapine | 0.023 | 0.004 | 0.000 | 0.000 |
| Alimemazine | 0.225 | 0.126 | 0.018 | 0.094 |
| Alprazolam | 0.186 | 0.067 | 0.016 | 0.174 |
| Amiodarone | 0.009 | 0.025 | 0.040 | 0.010 |
| Amitriptyline | 0.083 | 0.034 | 0.011 | 0.007 |
| Amlodipine | 0.051 | 0.080 | 0.089 | 0.064 |
| Amphetamine | 0.057 | 0.046 | 0.018 | 0.070 |
| Apixaban | 0.007 | 0.004 | 0.003 | 0.000 |
| Aripiprazole | 0.016 | 0.067 | 0.003 | 0.000 |
| Atenolol | 0.016 | 0.021 | 0.043 | 0.020 |
| Atomoxetine | 0.000 | 0.000 | 0.000 | 0.000 |
| Atropine | 0.005 | 0.017 | 0.001 | 0.000 |
| Baclofen | 0.002 | 0.000 | 0.002 | 0.000 |
| Bensoylecgonine | 0.021 | 0.017 | 0.002 | 0.013 |
| Biperiden | 0.011 | 0.050 | 0.001 | 0.000 |
| Bisoprolol | 0.016 | 0.046 | 0.037 | 0.010 |
| Bromazepam | 0.002 | 0.000 | 0.000 | 0.000 |
| Bupivacaine | 0.000 | 0.008 | 0.000 | 0.000 |
| Buprenorphine | 0.080 | 0.025 | 0.002 | 0.094 |
| Bupropion | 0.044 | 0.042 | 0.005 | 0.007 |
| Buspirone | 0.014 | 0.004 | 0.002 | 0.003 |
| Caffeine | 0.016 | 0.025 | 0.006 | 0.000 |
| Carbamazepine | 0.011 | 0.029 | 0.019 | 0.020 |
| Carisoprodol | 0.000 | 0.000 | 0.000 | 0.000 |
| Carvedilol | 0.000 | 0.008 | 0.000 | 0.000 |
| Chloroquine | 0.002 | 0.004 | 0.000 | 0.000 |
| Chlorpheniramine | 0.005 | 0.004 | 0.000 | 0.000 |
| Chlorprothixene | 0.011 | 0.017 | 0.000 | 0.000 |
| Citalopram | 0.126 | 0.105 | 0.069 | 0.077 |
| Clobazam | 0.000 | 0.004 | 0.000 | 0.000 |
| Clomethiazole | 0.005 | 0.004 | 0.000 | 0.000 |
| Clomipramine | 0.032 | 0.042 | 0.005 | 0.003 |
| Clonazepam / 7-amino-clonazepam | 0.092 | 0.034 | 0.012 | 0.104 |
| Clonidine | 0.000 | 0.008 | 0.000 | 0.000 |
| Clozapine | 0.011 | 0.013 | 0.002 | 0.000 |
| Cocaine | 0.011 | 0.008 | 0.001 | 0.003 |
| Codeine | 0.122 | 0.050 | 0.024 | 0.097 |
| Dabigatran | 0.000 | 0.008 | 0.000 | 0.000 |
| Dehydroaripiprazole | 0.007 | 0.042 | 0.000 | 0.000 |
| Desmethylalimemazine | 0.179 | 0.076 | 0.008 | 0.057 |
| Desmethylcitalopram | 0.108 | 0.059 | 0.044 | 0.043 |
| Desmethylclomipramine | 0.032 | 0.038 | 0.003 | 0.007 |
| Desmethylclozapine | 0.011 | 0.013 | 0.002 | 0.000 |
| Desmethyllevomepromazine | 0.018 | 0.034 | 0.000 | 0.003 |
| Desmethylmianserin | 0.011 | 0.008 | 0.001 | 0.000 |
| Desmethylmirtazapine | 0.152 | 0.113 | 0.082 | 0.090 |
| Desmethylolanzapine | 0.025 | 0.050 | 0.013 | 0.020 |
| Desmethylpromethazine | 0.057 | 0.046 | 0.007 | 0.020 |
| Desmetylsertraline | 0.078 | 0.063 | 0.039 | 0.040 |
| Dexmedetomidine | 0.000 | 0.004 | 0.000 | 0.000 |
| Dextromethorphan | 0.002 | 0.000 | 0.000 | 0.000 |
| Dextropropoxiphene | 0.016 | 0.004 | 0.000 | 0.003 |
| Dextrorphan | 0.000 | 0.000 | 0.000 | 0.000 |
| Diazepam | 0.172 | 0.126 | 0.041 | 0.130 |
| Dihydrocodeine | 0.002 | 0.004 | 0.000 | 0.000 |
| Diklofenac | 0.007 | 0.000 | 0.001 | 0.000 |
| Diltiazem | 0.002 | 0.013 | 0.002 | 0.000 |
| Diphenhydramine | 0.000 | 0.000 | 0.000 | 0.000 |
| Doxylamine | 0.000 | 0.000 | 0.000 | 0.000 |
| Duloxetine | 0.067 | 0.055 | 0.009 | 0.027 |
| EDDP | 0.037 | 0.017 | 0.000 | 0.003 |
| Edoxaban | 0.000 | 0.004 | 0.000 | 0.000 |
| Ephedrine | 0.007 | 0.025 | 0.008 | 0.000 |
| Ethanol | 0.359 | 0.185 | 0.386 | 0.639 |
| Ethylmorphine | 0.016 | 0.004 | 0.008 | 0.013 |
| Ethylphenidate | 0.005 | 0.008 | 0.000 | 0.000 |
| Etoricoxib | 0.011 | 0.008 | 0.003 | 0.000 |
| Felodipine | 0.000 | 0.000 | 0.000 | 0.000 |
| Fentanyl | 0.044 | 0.050 | 0.012 | 0.033 |
| Flecainide | 0.000 | 0.000 | 0.001 | 0.000 |
| Fluconazole | 0.002 | 0.021 | 0.002 | 0.003 |
| Flunitrazepam / 7-aminoflunitrazepam | 0.034 | 0.004 | 0.004 | 0.007 |
| Fluoxetine | 0.037 | 0.025 | 0.010 | 0.000 |
| Flupentixol | 0.002 | 0.008 | 0.002 | 0.000 |
| Fluphenazine | 0.000 | 0.000 | 0.000 | 0.000 |
| Fluvoxamine | 0.002 | 0.000 | 0.000 | 0.000 |
| Gabapentin | 0.051 | 0.042 | 0.011 | 0.020 |
| Glibenclamide | 0.000 | 0.004 | 0.000 | 0.000 |
| Glipizide | 0.000 | 0.004 | 0.000 | 0.000 |
| Haloperidol | 0.007 | 0.050 | 0.011 | 0.003 |
| Hydrocodone | 0.009 | 0.008 | 0.000 | 0.003 |
| Hydromorphone | 0.000 | 0.000 | 0.000 | 0.000 |
| Hydroxizine | 0.120 | 0.063 | 0.031 | 0.037 |
| Hydroxybupropione | 0.041 | 0.042 | 0.005 | 0.003 |
| Hydroxycarbazepine | 0.000 | 0.004 | 0.000 | 0.000 |
| Hydroxychloroquine | 0.002 | 0.000 | 0.001 | 0.000 |
| Hydroxyrisperidone | 0.032 | 0.029 | 0.014 | 0.003 |
| Isopropanol | 0.014 | 0.004 | 0.003 | 0.003 |
| Ketamine | 0.005 | 0.055 | 0.008 | 0.003 |
| Ketobemidone | 0.009 | 0.004 | 0.002 | 0.000 |
| Ketoprofen | 0.000 | 0.004 | 0.000 | 0.000 |
| Labetalol | 0.002 | 0.004 | 0.002 | 0.000 |
| Lacosamide | 0.002 | 0.013 | 0.000 | 0.000 |
| Lamotrigine | 0.051 | 0.059 | 0.018 | 0.010 |
| Lercanidipine | 0.002 | 0.004 | 0.000 | 0.000 |
| Levetiracetam | 0.002 | 0.038 | 0.005 | 0.003 |
| Levomepromazine | 0.034 | 0.050 | 0.001 | 0.010 |
| Lidocaine | 0.005 | 0.025 | 0.007 | 0.000 |
| Loperamid | 0.014 | 0.013 | 0.001 | 0.000 |
| Lorazepam | 0.009 | 0.004 | 0.000 | 0.000 |
| Maprotiline | 0.000 | 0.004 | 0.000 | 0.000 |
| MDA | 0.009 | 0.004 | 0.000 | 0.000 |
| MDMA | 0.011 | 0.008 | 0.001 | 0.000 |
| Melperone | 0.002 | 0.004 | 0.000 | 0.000 |
| Memantine | 0.000 | 0.004 | 0.001 | 0.000 |
| Mepivacaine | 0.000 | 0.008 | 0.001 | 0.000 |
| Meprobamate | 0.000 | 0.000 | 0.000 | 0.000 |
| Metamphetamine | 0.005 | 0.008 | 0.000 | 0.000 |
| Metformin | 0.021 | 0.017 | 0.012 | 0.007 |
| Methadon | 0.085 | 0.038 | 0.002 | 0.077 |
| Methanol | 0.005 | 0.000 | 0.000 | 0.000 |
| Methylphenidate | 0.053 | 0.046 | 0.005 | 0.023 |
| Methylscopolamine | 0.002 | 0.000 | 0.000 | 0.000 |
| Metoklopramid | 0.018 | 0.042 | 0.003 | 0.010 |
| Metoprolol | 0.094 | 0.126 | 0.131 | 0.060 |
| Metronidazole | 0.002 | 0.008 | 0.000 | 0.000 |
| Mianserin | 0.016 | 0.013 | 0.001 | 0.003 |
| Midazolam | 0.002 | 0.038 | 0.006 | 0.003 |
| Mirtazapine | 0.163 | 0.130 | 0.091 | 0.114 |
| Moclobemide | 0.000 | 0.000 | 0.000 | 0.000 |
| Morphine | 0.152 | 0.084 | 0.038 | 0.134 |
| N-desethylamiodarone | 0.005 | 0.021 | 0.014 | 0.010 |
| Naproxene | 0.011 | 0.013 | 0.010 | 0.010 |
| Nifedipin | 0.000 | 0.000 | 0.000 | 0.000 |
| Nitrazepam / 7-amino-nitrazepam | 0.037 | 0.063 | 0.008 | 0.017 |
| Norbuprenorphine | 0.067 | 0.021 | 0.002 | 0.080 |
| Norclobazam | 0.000 | 0.004 | 0.000 | 0.000 |
| Nordazepam | 0.184 | 0.139 | 0.040 | 0.151 |
| Norephedrin | 0.002 | 0.017 | 0.000 | 0.000 |
| Norfluoxetine | 0.039 | 0.025 | 0.011 | 0.003 |
| Nortriptyline | 0.067 | 0.021 | 0.004 | 0.003 |
| Norverapamil | 0.005 | 0.004 | 0.002 | 0.000 |
| Noscapine | 0.002 | 0.000 | 0.000 | 0.000 |
| O-desmethyltramadol | 0.087 | 0.034 | 0.016 | 0.020 |
| O-desmethylvenlafaxine | 0.087 | 0.063 | 0.024 | 0.030 |
| Olanzapine | 0.046 | 0.084 | 0.027 | 0.030 |
| Orphenadrine | 0.000 | 0.000 | 0.000 | 0.000 |
| Oxazepam | 0.126 | 0.105 | 0.039 | 0.060 |
| Oxicodone | 0.129 | 0.067 | 0.036 | 0.084 |
| Papaverine | 0.000 | 0.000 | 0.000 | 0.000 |
| Paracetamol | 0.172 | 0.181 | 0.124 | 0.057 |
| Paroxetine | 0.016 | 0.038 | 0.008 | 0.007 |
| Perphenazine | 0.009 | 0.038 | 0.001 | 0.003 |
| Pethidine | 0.005 | 0.000 | 0.000 | 0.000 |
| Phenazepam | 0.002 | 0.000 | 0.000 | 0.000 |
| Phenazone | 0.000 | 0.000 | 0.000 | 0.000 |
| Phenytoin | 0.000 | 0.013 | 0.001 | 0.000 |
| Pindolol | 0.000 | 0.000 | 0.000 | 0.000 |
| Pregabalin | 0.186 | 0.063 | 0.013 | 0.084 |
| Primidone | 0.000 | 0.000 | 0.000 | 0.000 |
| Promethazine | 0.069 | 0.059 | 0.014 | 0.037 |
| Propiomazine / dihydropropiomazine | 0.230 | 0.126 | 0.050 | 0.124 |
| Propranolol | 0.057 | 0.021 | 0.005 | 0.010 |
| Pseudoephedrine | 0.000 | 0.004 | 0.000 | 0.000 |
| Quetiapine | 0.046 | 0.042 | 0.006 | 0.020 |
| Quinine | 0.002 | 0.008 | 0.000 | 0.000 |
| Reboxetine | 0.000 | 0.008 | 0.000 | 0.000 |
| Reduced haloperidol | 0.014 | 0.055 | 0.010 | 0.003 |
| Risperidone | 0.025 | 0.021 | 0.006 | 0.000 |
| Ritalic acid | 0.060 | 0.050 | 0.005 | 0.023 |
| Rivaroxaban | 0.000 | 0.004 | 0.000 | 0.000 |
| Ropivacaine | 0.000 | 0.000 | 0.000 | 0.000 |
| S-methyldihydroziprasidone | 0.005 | 0.000 | 0.000 | 0.000 |
| Sertraline | 0.101 | 0.076 | 0.049 | 0.037 |
| Sotalol | 0.005 | 0.008 | 0.002 | 0.000 |
| Tamoxifen | 0.009 | 0.004 | 0.000 | 0.000 |
| Tapentadol | 0.002 | 0.008 | 0.000 | 0.000 |
| Temazepam | 0.023 | 0.029 | 0.001 | 0.000 |
| Theophylline | 0.002 | 0.000 | 0.000 | 0.000 |
| Thioridazine | 0.000 | 0.004 | 0.000 | 0.000 |
| Topiramate | 0.009 | 0.017 | 0.002 | 0.000 |
| Tramadol | 0.097 | 0.038 | 0.018 | 0.020 |
| Triazolam | 0.000 | 0.000 | 0.000 | 0.000 |
| Trihexyphenidyl/bezshexol | 0.002 | 0.004 | 0.000 | 0.000 |
| Trimethoprim | 0.000 | 0.017 | 0.001 | 0.000 |
| Trimipramin | 0.000 | 0.000 | 0.000 | 0.000 |
| Venlafaxine | 0.094 | 0.059 | 0.023 | 0.037 |
| Verapamil | 0.005 | 0.004 | 0.002 | 0.000 |
| Vortioxetine | 0.011 | 0.013 | 0.002 | 0.000 |
| Warfarin | 0.002 | 0.034 | 0.020 | 0.003 |
| Zaleplon | 0.000 | 0.000 | 0.000 | 0.000 |
| Ziprasidone | 0.005 | 0.000 | 0.000 | 0.000 |
| Zolpidem | 0.129 | 0.063 | 0.016 | 0.060 |
| Zonisamide | 0.002 | 0.004 | 0.000 | 0.000 |
| Zopiclone | 0.340 | 0.160 | 0.094 | 0.161 |
| Zuclopenthixol | 0.018 | 0.050 | 0.009 | 0.007 |
